# Brain structure comparison among Parkinson disease, essential tremor, and healthy controls using 7T MRI

**DOI:** 10.1097/MD.0000000000038139

**Published:** 2024-05-10

**Authors:** Hyeong Cheol Moon, Aryun Kim, Young Seok Park

**Affiliations:** a Department of Neurosurgery, Gamma Knife Icon Center, Chungbuk National University Hospital, Cheongju, Republic of Korea; b Department of Neurology, Chungbuk National University Hospital, Chungbuk National University College of Medicine, Cheongju, Republic of Korea; c Department of Neurosurgery, Chungbuk National University College of Medicine, Cheongju, Republic of Korea.

**Keywords:** 7T MRI, brain structure, essential tremor, Parkinson disease

## Abstract

Both Parkinson disease (PD) and Essential tremor (ET) are movement disorders causing tremors in elderly individuals. Although PD and ET are different disease, they often present with similar initial symptoms, making their differentiation challenging with magnetic resonance imaging (MRI) techniques. This study aimed to identify structural brain differences among PD, ET, and health controls (HCs) using 7-Tesla (T) MRI. We assessed the whole-brain parcellation in gray matter volume, thickness, subcortical volume, and small regions of basal ganglia in PD (n* = *18), ET (n = 15), and HCs (n = 18), who were matched for age and sex. Brain structure analysis was performed automatic segmentation through Freesurfer software. Small regions of basal ganglia were manually segmented by ITK-SNAP. Additionally, we examined the associations between clinical indicators (symptom duration, unified Parkinson diseases rating scale (UPDRS), and clinical rating scale for tremor (CRST)) and brain structure. PD showed a significant reduction in gray matter volume in the postcentral region compared to ET. ET showed a significant reduction in cerebellum volume compared to HCs. There was a negative correlation between CRST scores (B and C) and gray matter thickness in right superior frontal in ET. This study demonstrated potential of 7T MRI in differentiating brain structure differences among PD, ET, and HCs. Specific findings, such as parietal lobe atrophy in PD compared to ET and cerebellum atrophy in ET compared to HCs, the importance of advanced imaging techniques in accurately diagnosing and distinguishing between movement disorders that present with similar initial symptoms.

## 1. Introduction

Parkinson disease (PD) and Essential tremor (ET) are prevalent movement disorders in the elderly. Due to their similarities in the early stages, they are often misdiagnosed since because they are challenging to differentiate both clinically and radiologically. PD primarily affects the dopaminergic neurons of the substantia nigra (SN). The symptoms of PD vary among patients and can include tremor, bradykinesia, rigid muscles, and loss automatic movement. In contrast, ET is one of the most common tremor disorders. Which has been considered a monosymptomatic disease characterized exclusively action tremor.^[1]^ Both PD and ET can present with various types of tremors,^[2]^ and tremor has occasionally been reported as a symptom of normal pressure hydrocephalus.^[3]^ Despite these diseases being distinct entities, their symptoms related to movement disorders often overlap. Treatment options for patients can be treated medications, deep brain stimulation, and thalamotomy.^[4,5]^ Diagnosis primarily relies on clinical examination of tremor features, positron emission tomography, and pathology test that help distinguish between 2 diseases.^[1,6–8]^ Imaging studies, ET showed that have a trend toward slightly lower striatal binding ratios using I-ioflupane single photon emission computed tomography compared to PD and HC.^[9–11]^ Another imaging studies showed brain structural changes with automated segmentation method.^[2,12,13]^ However, these studies were investigated using 1.5 Tesla (T) magnetic resonance imaging (MRI). Recently, ultra-high filed (magnetic field ≥ 7T) has been increased attention compared to 1.5T MRI or 3T MRI.^[14,15]^ The development of 7T MRI quietly advanced, increased inherent MR sensitivity and signal-to-noise (SNR), and approved the Food and Drug Administration on clinical fields. Movement disorders could be related to brain structure changes. 7T MRI could be potential to detect brain structure changes. Utilizing 7T MRI to obtain high-resolution structure images of the brain, and segmented these images, allows for a detailed confirmation of brain structure differences. Morphological changes and pathological mechanism of gray matter in the brain are associated with both PD^[16–18]^ and ET.^[19]^ The characteristic of gray matter can include regions of demyelination, apoptotic neurons and atrophy of cortical volume and thickness.^[20–22]^

In this study, we performed automated segmentation techniques to parcellate brain structures, ensuring precision through corrected reconstructions in high-resolution imaging. We conducted a comparative analysis of cortical and subcortical differences among 3 groups based on the general linear model. Furthermore, manual segmentation was applied to small regions of basal ganglia. We examined the correlation between brain structures and clinical indicators.

## 2. Methods

### 2.1. Participants

A total of 51 participants, comprising patients diagnosed with PD (n = 18) and ET (n = 15) and HCs (n = 18), were enrolled in this study. We examined the clinical indicators, including gender, age, symptom duration, tremor laterality, Movement Disorder Society-Unified Parkinson Disease Rating Scale (MDS-UPDRS) for PD^[23]^ and Clinical Rating Scale for Tremor (CRST) for ET.^[24]^ We considered the side of illness in relation to the ipsilateral and contralateral areas. All study protocols were approved by the Institutional Review Board (IRB number: 2016-12-009-005) from the Chungbuk National University Hospital, and informed consent was obtained from all participants.

### 2.2. MRI acquisition

All participants were acquired images using a 7T MRI (Philips Healthcare, Cleveland, OH) with 32-channel head coil (Nova Medical, Wilmington, MA). Three-dimensional anatomical brain scans were acquired using magnetization prepared rapid acquisition gradient echo sequence-induced T1-weighted image with the following setting: repetition time (TR) = 4.4 milliseconds (ms), echo time (TE) = 2.2ms, slice thickness = 0.5mm, acquisition time (TA) = 357 seconds (s). The turbo spin-echo sequence-induced T2-weighted image with the following setting: TR = 7000ms, TE = 72ms, slice thickness = 2mm, TA = 308s.

### 2.3. Image analysis

**Figure. 1.**
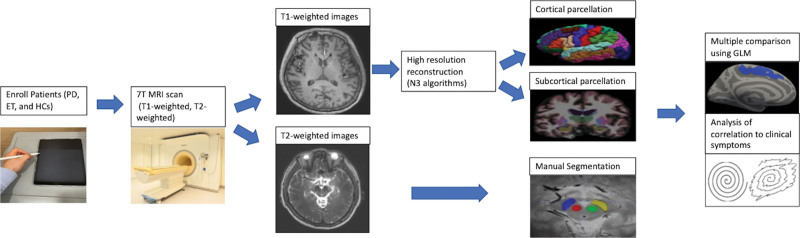
Flowchart of the study protocol.

High-resolution images required modifications to the default reconstruction process using the nonparametric, nonuniform intensity, and normalization (N3) algorithm for inhomogeneity correction for 7T MRI.^[25]^ The cortical reconstruction and segmentation were conducted using Freesurfer software (Version 6.0). Previous publications of high-resolution data (<1 mm) were used to modify the default stream and inhomogeneity correlation by division before data processing.^[26,27]^ The FreeSurfer pipline performed cortical and subcortical segmentation, included the removal of non-brain tissue as part of its standard protocol for high-resolution data. Gray matter cortex was parcellated with allocations for labeling in accordance with Desikan-Killiany-Tourville neuroanatomical atlas, segmenting the cortex into 31 distinct regions per hemisphere. Gray matter volume and thickness measurements were derived at the vertex of the pial surface. The general linear model (GLM) was used to assess each participant vertex to vertex for cortical thickness and volume, adjusted for age and gender factors using FreeSurfer mri_glmfit. (http://surfer.nmr.mgh.harvard.edu/fswiki/mri_glmfit). A cluster-wise correction was executed Fressurfer mri_glmfirt-sim (http://surfer.nmr.mgh.harvard.edu/fswiki/FsTutorial/GroupAnalysis) tool to precompute a Z Monte Carlo simulation, setting a vertex threshold of 2 (*P* value < .01). Subcortical volumes were quantified using FreeSurfer and small regions of basal ganglia were manually segmented using ITK-SNAP Software (http://www.itksnap.org). The cerebellum was classified as a “subcortical volume” based on its anatomical location beneath the cerebral cortex. ITK-SNAP provides manual segmentation using active contouring methods, as well as semi-automatic and image navigation.^[28]^ We executed manually slice-by-slice segmentation by neurosurgeon, neurologist, radiologist, and medical physicist each with over 5 years of experience using the software. All study protocol is shown in Figure 1.

### 2.4. Statistical analysis

Statistical analyses were conducted using GraphPad Prism 9 (GraphPad Software, San Diego, CA) and SPSS (Version 21., SPSS Inc., Chicago, IL). Age differences were analyzed using nonparametric analysis of variance of Kruskal-Wallis test, followed by Dunn multiple comparison post hoc test. The Chi-square test was employed to assess gender differences. A multivariate analysis of variance for statistical computations performed on subcortical regions differences controlled with age and sex as covariates. A significance threshold was established at *P* value < .05.

## 3. Results

### 3.1. Clinical characterization and demographic

Details of the participants characteristics are presented in Table 1. There were no significant differences in age, gender, and symptom duration in PD, ET, and HCs. Enrolled patients with tremor symptoms were categorized as right-side, left-side, or bilateral. The mean MDS-UPDRS for parts I, II, III, and IV were 3, 11, 28, and 8, respectively. The mean CRST scores for parts A, B, and C were 17, 4, and 9, respectively.

**Table 1:** Demographic and clinical characteristics of PD patients, ET patients, and HCs.

	PD (n = 18)	ET (n = 15)	HCs (n = 18)	*P* value
Median age (yr)*	64	64	56	NS
Gender (males/females)†	7/11	9/6	7/11	NS
Symptom duration (yr)‡	3.5	4.2	N/A	NS
Tremor Laterality (Left/Right/Bilateral)	5/5/8	0/4/11	N/A	N/A
MDS-UPDRS I/II/III/IV	3/11/28/8	N/A	N/A	N/A
CRST A/B/C	N/A	17/4/9	N/A	N/A

CRST = clinical rating scale for tremor, ET = essential tremor, HC = healthy controls, MDS-UPDRS = movement disorder society-unified Parkinson disease rating scale, N/A = not applicable, NS = not significant, PD = Parkinson disease.

* Age was compared using nonparametric analysis of variance (ANOVA) of Kruskal-Wallis test followed by Dunn multiple comparison post hoc test

† Sex was accessed using Chi-square

‡ Disease duration was compared using nonparametric unpaired *t-test* followed by Mann-Whitney test

### 3.2. Comparison of gray matter thickness and volume

**Figure. 2.**
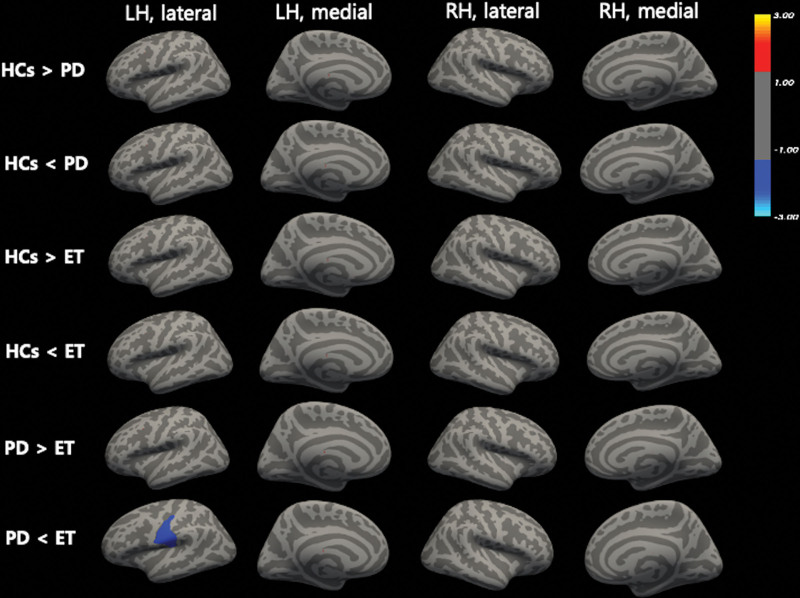
Comparison of gray matter volume differences among PD, ET, and HCs. ET = essential tremor, HCs = health controls, PD = Parkinson disease.

There were no significant differences in gray matter thickness among PD, ET and HCs when analyzed using the GLM model with FreeSurfer Group Descriptor, adjusted for age and sex as covariates. The gray matter volume of postcentral regions showed atrophy compared to those with ET (Fig. 2 and Table 2).

**Table 2:** Gray matter volume differences among PD, ET, and HCs.

Contrast	Hemisphere	Measure	Max *t*-stat	Association (Positive or Negative)	Cluster size (mm^2^)	Tal X	Tal Y	Tal Z	CWP	Anatomy	*P* value
PD vs ET	left	Volume	−3.50	Negative	701.76	−41.9	−15.2	30.1	0.03	Postcentral	**P < .01

CWP = cluster-wise probability, Tal = Talairach X, Y, and Z coordinates, t-stat = t-statistic.

### 3.3. Comparison of subcortical volume

Differences in subcortical volumes for all participants are presented in Table 3. Post hoc comparisons revealed significant differences in cerebellum cortex between ET and HCs. Each cerebellar hemisphere was analyzed separately using the methods provided by Freesurfer software, and this approach was applied with the clinical asymmetry of the patients in mind. No significant differences were observed in other subcortical regions among PD, ET, and HCs.

**Table 3:** Subcortical volume differences among PD, ET, and HCs.

Structures (mm^3^)	PD	ET	HCs	*P* value†	Post hoc test‡		
					PD vs HCs	PD vs ET	ET vs HCs
Cerebellum-White-Matter	16157.28 ± 9037.12	16398.88 ± 12181.12	17839.06 ± 9219.08	NS	NS	NS	NS
Cerebellum Cortex	52635.95 ± 18624.95	47721.45 ± 16573.55	55641.55 ± 16089.84	NS	NS	NS	*0.013
Thalamus	15725.22 ± 2131.78	15942.69 ± 1160.99	15531.60 ± 1351.12	NS	NS	NS	NS
Caudate	5799.85 ± 984.08	6364.81 ± 760.63	6049.81 ± 863.83	NS	NS	NS	NS
Putamen	8886.71 ± 717.97	9465.39 ± 1402.88	9301.49 ± 1027.34	NS	NS	NS	NS
Pallidum	4007.31 ± 623.53	4152.66 ± 688.97	4152.65 ± 433.46	NS	NS	NS	NS
Hippocampus	5966.025 ± 1417.47	6300.96 ± 986.27	6805.61 ± 775.85	NS	NS	NS	NS
Amygdala	2285.56 ± 632.25	2665.05 ± 540.03	2720.12 ± 393.02	NS	NS	NS	NS
Accumbens	639.29 ± 147.47	625.41 ± 173.34	756.48 ± 141.72	NS	NS	NS	NS
White matter hypointensities	6726.11 ± 6873.27	3840.36 ± 448.38	5019.07 ± 2175.83	NS	NS	NS	NS
Brain stem	16901.81 ± 2974.28	16677.34 ± 2764.37	17876.05 ± 1988.40	NS	NS	NS	NS
CSF	1289.41 ± 424.01	1374.99 ± 302.27	1133.30 ± 239.15	NS	NS	NS	NS

CSF = Cerebrospinal fluid, ET = Essential tremor, HCs = Healthy controls, NS = No significantly, PD = Parkinson disease.

† *P* value of the multivariate analysis of variance (MANOVA) controlled with age and sex as covariates, *P < .05, **P < .01.

‡ Post hoc test analysis was performed with Bonferroni multiple comparisons.

### 3.4. Comparison of small regions of basal ganglia

We measured the volume of small regions in basal ganglia (Caudate, Globus Pallidus, Putamen, Substantia nigra, Subthalamic nucleus, and ventral pallidum). However, there were no significant differences among PD, ET, and HCs.

### 3.5. Correlation between clinical indicators and brain structure

There is no correlation between clinical indicators and brain structure in PD. CRST scores were negatively correlated with superior frontal thickness (Table 4). There were no correlations observed for other clinical indicators in ET.

**Table 4:** Correlation between clinical indicators and brain structure in ET patients.

Patients (PD or ET)	Indicators	Hemisphere	Measure	Max *t*-stat	Association (Positive or Negative)	Cluster size (mm^2^)	Tal X	Tal Y	Tal Z	CWP	Anatomy
ET	CRST B	Right	Thickness	−3.753	Negative	599.20	9.7	19.2	60.4	0.039	Superior frontal
ET	CRST C	Right	Thickness	−3.620	Negative	633.59	8.3	14.8	59.4	0.029	Superior frontal

CRST = clinical rating scale for tremor, CWP = cluster-wise probability, ET = essential tremor, Tal = Talairach X, Y, and Z coordinates, t-stat = t-statistic.

## 4. Discussion

This study aimed to investigate clinical indicators and brain structure differences among PD, ET, and HCs using 7T MRI. We first demonstrate the clinical application of 7T MRI in diagnosing these groups. Among these groups, PD showed parietal lobe atrophy compared ET, while ET showed cerebellum atrophy compared to HCs, and negative correlation between CRST scores and frontal atrophy.

PD is characterized clinically by slowing progressive symptoms an pathologically by the degeneration of pigment neuromelanin-bearing cells of substantia nigra.^[29]^ PD exhibit bradykinesia and at least 1 of 3 other features: rigidity, resting tumor, or postural instability.^[30,31]^ While advanced PD is relatively easy to diagnose, distinguishing early-stage PD from ET can often be challenging.^[6,32]^ The parietal lobe plays a crucial role in integrating sensory information and is involved in spatial sense and navigation. In our study, PD showed parietal atrophy compared to ET. While the parietal lobe is not traditionally associated with the generation of tremors in PD, changes or atrophy in this region might be correlated with disease progression. The connection between parietal lobe atrophy and PD symptoms may not be direct, but rather indicative of a more widespread neurodegenerative process that affects multiple brain regions, including both the motor and cognitive domains.

ET is a progressive neurological disorder characterized by action tremors. Its exact cause remains unclear but appears to have a familial.^[33,34]^ ET can be diagnosed at any age, from younger individuals to advanced ages, and is commonly observed in the hands and arms. Around one-half of ET cases are associated with genetic mutations, typically following an autosomal dominant trasmission.^[35,36]^ Previous studies have shown Purkinje cell loss and axonal swelling in cerebellum as neurogenerative features in ET.^[2–45]^ ET showed Recent studies have reported evidence related to Leucine-rich repeat and Ig domain containing 1 gene^[37,38]^ and GABA receptors^[37,38]^ in the cerebellum of ET. Imaging studies have also suggested involvement of the cerebellothalamocortical circuit.^[39,40]^ In our study, ET showed cerebellum volume reduction, although thalamus volume differences compared to PD and HC were not evident. While some studies agree with structural changes and cell loss in the cerebellum,^[41,42]^ others have reported controversial results.^[19,43]^ There are several possible reasons to support brain structure changes in ET. Firstly, the progression of tremor symptoms is faster and more sever in ET, likely resulting in greater cellular-level degeneration.^[44]^ Secondly, whole-brain analysis allows for the exploration of widespread networks but may risk ignoring certain regions.^[6]^ 7T MRI data provides high contrast and resolution but requires careful interpretation and analysis due to potential variations. Lastly, ET revealed the widespread cortical abnormalities based on aboard alteration.^[43,45]^

3T MRI are commonly used in clinical fields. However, high-resolution 7T imaging has the potential to improve the detection and characterization of abnormalities associated with various neurological disorders, brain tumors, and neuropsychiatric disorders.^[46–48]^ 7T MR imaging offers finer anatomical details, increased lesion visibility, and susceptibility effects for better brain visualization.^[49,50]^ The effective resolution of 7T MRI in humans can be significantly enhanced using prospective motion correction and longer scan times.^[51]^ Most current research focuses on improving hardware and sequence optimization of diagnostic examinations due to issues like inhomogeneous transmit fields, increased artifacts, and specific absorption rate limitations.^[14,52]^ In dealing with inhomogeneity, 7T MRI can negatively impact structural analysis. The N3 method can be a useful tool for bias field correction to address this issue and improve image segmentation.^[53]^ Although high-tesla MRI generally provides better results than low-field tesla MRI, the fully potential of high-tesla MRI has yet to be fully demonstrated.^[54]^ Recently, the FDA cleared the first 7T MRI device, the Magnetom Terra, for use in the USA. Theoretical results and 32-channel coil data have shown a SNR of approximately 50 values in the cortex and vertex of the head, which is lower than reported 70 values.^[55]^ Moreover, 32-channel data can significantly increase SNR values in 7T functional MRI, which is valuable for achieving high spatial resolution.^[56]^ In our study, we employed 32-channel head coils, which may provide optimized image sequences for clinical and technical applications in 7T MRI.

The present study has several limitations. Firstly, the number of PD, ET, and HCs was relatively small; therefore, future studies with larger sample sizes are needed. Secondly, this cross-sectional study cannot establish cause-and-effect relationships or elucidate pathological mechanisms. Future research should involve longitudinal observations of changes in ET and PD patients. To ensure safety in 7T MRI, advanced PD and elderly individuals were excluded, as were patients with existing health problems such as surgical prostheses or dental implants. It is possible that these exclusions were not sufficient to demonstrate differences in neurodegenerative changes.

## 5. Conclusions

We identified the brain structure differences among ET, PD, and HCs using 7T MRI. High-resolution images were processed using the N3 algorithm for MR inhomogeneity correction. Our findings revealed that PD showed parietal lobe atrophy compared to ET. ET revealed significant cerebellum volume reduction compared to HCs. There was negative correlation between CRST scores and frontal in ET. This research is consequential as it discerns differences in neurodegenerative structural changes among the 3 groups by observing brain structure alterations through 7T MRI. It unveiled distinctions between PD and ET, enhancing our understanding of these conditions. PD and ET are prevalent movement disorders in the elderly, often subject to misdiagnosis and exhibiting diverse progression patterns. Thus, discerning the progression of brain lesions is crucial for accurate diagnosis and effective treatment planning.

## Author contributions

**Conceptualization:** Young Seok Park.

**Data curation:** Hyeong Cheol Moon, Young Seok Park, Aryun Kim.

**Funding acquisition:** Young Seok Park.

**Methodology:** Young Seok Park, Aryun Kim.

**Resources:** Aryun Kim.

**Software:** Hyeong Cheol Moon.

**Validation:** Hyeong Cheol Moon.

**Visualization:** Young Seok Park.

**Writing – original draft:** Hyeong Cheol Moon.

**Writing – review & editing:** Young Seok Park.
